# Prevalence and factors affecting the utilization of antenatal care in rural areas of Southwestern Ethiopia

**DOI:** 10.1186/s12884-021-04362-8

**Published:** 2022-01-14

**Authors:** Assaye Belay, Tessema Astatkie, Solomon Abebaw, Bekele Gebreamanule, Wegayehu Enbeyle

**Affiliations:** 1 Department of Statistics, Mizan-Tepi University, Tepi, Ethiopia; 2 Faculty of Agriculture, Dalhousie University, Truro, NS B2N 5E3 Canada; 3 Department of Biology, Injibara University, Injibara, Ethiopia

**Keywords:** Antenatal care, Odds ratio, Binary logistic regression model, Rural zone, Women

## Abstract

**Background:**

Antenatal care (ANC) is a health care intervention intended to ensure the safety of pregnancy. According to the World Health Organization, at least four ANC visits are recommended for a healthy pregnancy. However, whether this recommended number of visits was followed or not in the rural areas of Southwestern Ethiopia is not known. Therefore, the study aimed to investigate the prevalence of, and the associated factors of ANC utilization by pregnant women in the rural areas of Southwestern Ethiopia.

**Methods:**

A community-based cross-sectional study design was used in three rural zones. The data were collected from n = 978 women through a structured questionnaire with face-to-face interview. The collected data were analyzed using descriptive statistics and a multiple binary logistic regression model.

**Results:**

The results showed that 56% of women made the recommended minimum number of ANC visits and the remaining 44% of them underutilized the ANC service. The multiple binary logistic regression model identified zone, marital status of the woman, educational level of the husband, occupation of the husband, knowledge of danger signs of pregnancy, birth interval, source of information, timely visits, and transportation problem to be statistically significant factors affecting the prevalence of ANC visit utilization of women. Bench Maji zone had smaller odds ratio of ANC visit prevalence as compared to Kaffa zone. Women who lived in the rural area of Sheko zone are 2.67 times less likely to utilize ANC visit than those who lived in the rural area of Kaffa zone keeping other variables constant.

**Conclusion:**

The study results highlight the need to increase the number of ANC visits, and the importance of using an appropriate model to determine the important socio-demographic factors that ANC service providers shall focus on to improve the health of the unborn baby and the mother during pregnancy.

## Background

Antenatal care (ANC) is an important care provided to pregnant women to improve the health of the unborn baby and the mother [1]. According to the recommendation of World Health Organization (WHO), every pregnant woman should start taking ANC before 12 weeks of pregnancy, and that any healthy pregnant woman should receive at least four ANC visits to reduce maternal death due to pregnancy complications [2, 3].

The utilization of ANC services by rural area women during pregnancy is constrained by different problems [4]. A cross-sectional study from Nigeria showed that the national average of ANC utilization rate is 61%, of which 81% of them had ≥4 visits [5]. Tolera et al. [6] reported that a very high proportion of women had attended below the recommended (≥4 visits) ANC visits in rural areas of western Ethiopia. In Nigeria, a comparative study of rural and urban residences based on the 2013 demographic and health survey showed that only 39% of rural women had four or more ANC visits as compared to 78% of urban women [7]. However, in rural areas of Ethiopia, more women have delayed initiation of ANC visits compared to women in urban areas [8]. Early launch of ANC is beneficial to detect and treat complications during pregnancy before it is too late [9]. However, majority of the womenin Ethiopia initiate ANC late [10].

Most maternal ANC studies in Ethiopia are based on data from urban areas and health facilities, but not from rural communities. Therefore, the objective of this study was to assess whether the number of ANC visits meets the WHO recommendation, and to identify the factors affecting it among ANC service users in rural area of southwest Ethiopia.

## Methods

### Study area

**Fig. 1:**
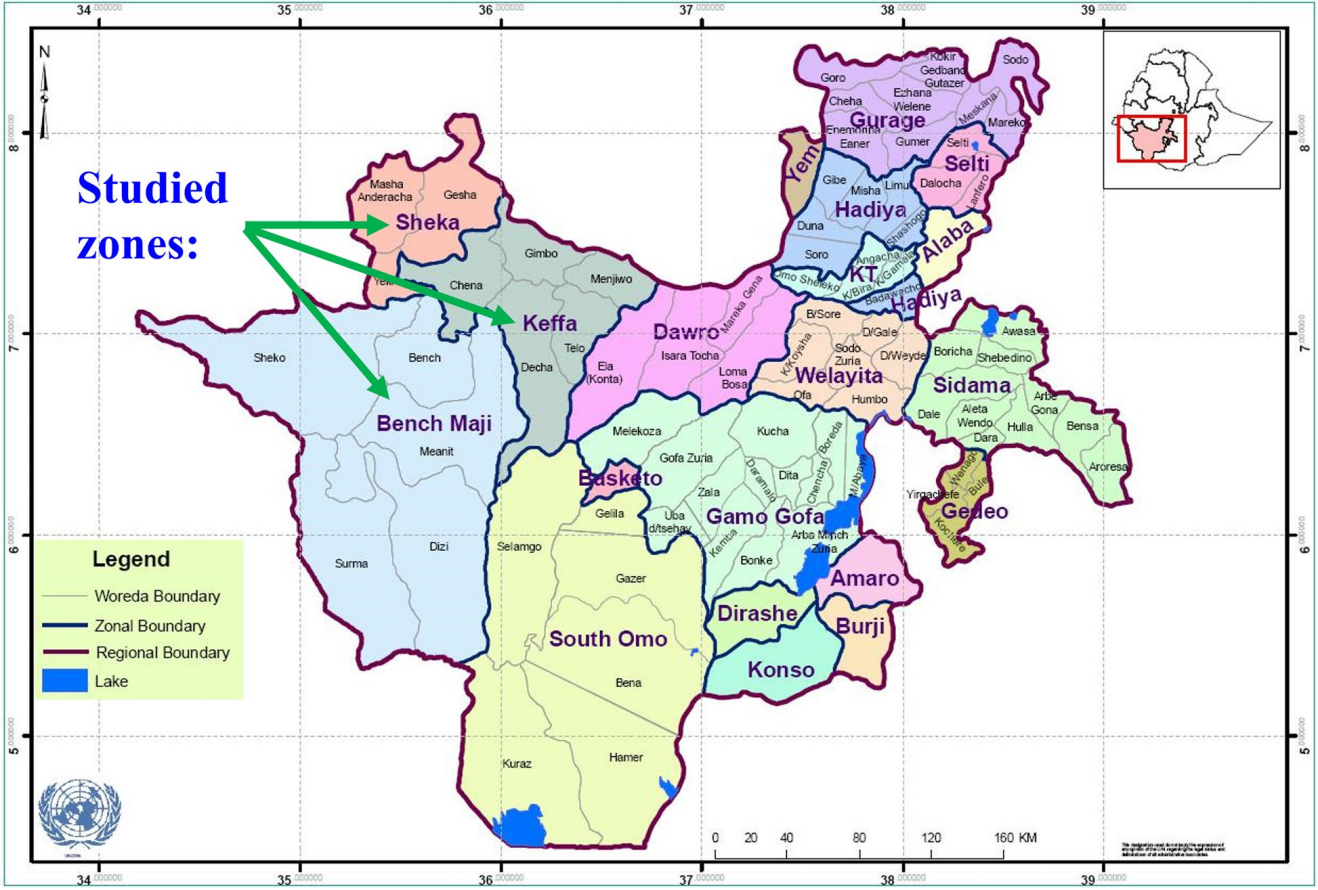
Location of the study area (Kaffa zone, Sheka zone, and Bench Maji zone)

The study was conducted in Kaffa, Sheka, and Bench Maji zones of Southern Nations, Nationalities, and Peoples’ Region (SNNPR). These three zones are in Southwestern Ethiopia (Fig. 1).

The capital city of Kaffazone (Bonga) is 571 km south-west of Hawassa (the capital city of SNNPR) and 405 km south of Addis Ababa (the capital city of Ethiopia). Kaffa zone has 297 health posts, 45 health centers, one general hospital, one primary hospital, and 7 Woreda (equivalent to county) health offices. According to the 2007 census conducted by the Central Statistical Agency (CSA) [11], the population of Kaffa zone is 871,984, of which 442,166 are females. Among the females, 32,182 (17,783 are in reproductive age [15-49]) live in urban areas and the remaining 409,984 (180,046 are in reproductive age) live in rural areas.

Sheka zone has 57 health posts, one general hospital, one primary hospital, one health center, and 5 Woreda health offices. The capital city of Sheka zone is Masha, and it is 951 km from Hawassa and 676 km from Addis Ababa. The population of the zone is 199,314 [11], of which 98,255 are females (48,939 in reproductive age group). Among all females, 16,482 of them live in urban areas (9,568 in reproductive age group), and 81,773 of them live in rural areas (39,371 in reproductive age group).

Bench Maji zone has 134 health posts, 27 health centers, one teaching hospital, one primary hospital, and seven Woreda health offices. The population ofthe zone is 652,531 [11], of which 329,183 are females, and 157,740 of them are in reproductive age (15-49) group. Among all reproductive females, 19,626 live in urban areas and 138,114 of them live in rural areas.

### Sampling procedure and data collection method

Primary data were collected from a community-based cross-sectional survey conducted in the three rural zones (Kaffa, Sheka,and Bench Maji) that are in the western part of SNNPR. These three zones have 28 (13, 3, and 12 in Kaffa, Sheka,and Bench Maji zones, respectively) woredas (counties) that are considered internally heterogenous and externally homogenous, which makes them appropriate to be considered as clusters. A three-stage sampling method was used, where 3 woredas were randomly selected in the first stage, and 2 kebeles (equivalent to districts) were randomly selected from each of the 3 Woredas in the second stage. The 6 randomly selected kebeles are Haro, Kushit, Ermo, Fide, Yeba, and Ermich. The sample size (n = 978) was determined as described in Umulisa [12]. Among the women living in these 6 kebeles and who gave birth during the last five years, n = 978 of them were randomly selected, and a structured questionnaire with face-to-face interview was used to collect the data.

### Study variables

The dependent variable was ANC visits, and its binary values were either at least 4 ANC visits (≥4 visits) or less than 4 ANC visits. The threshold for the values was based on the recommendation of ANC visits by WHO for healthy pregnancy of women [2]. The independent variables, identified based on the literaturewere Zone, Marital status of the woman, Religion, Educational level of the woman, Educational level of the husband, Occupation of the mother, Occupation of the husband, Was there abortion? Iron-folate supplementation use, Decision maker, Knowledge of danger signs of pregnancy (e.g., vomiting, heavy headache, and depression), Birth interval, Source of information, Volunteering, Timely visit, Transportation problem, Satisfaction of previous visits, Distance, and Age of the woman; and their values were collected from each respondent using astructured questionnaire.

### Statistical analyses

Contingency tables of socio-demographic categorical variables and ANC service utilization of women who gave birth during the past five years were produced to show the percentages of the respondents falling in the different cells of the table. Based on a preliminary chi-square test of independence, the categorical variables whose association with the response variable (ANC service utilization) showed a p-value of ≤ 0.25 were included in the multiple logistic regression model analysis that was used to determine the probability that each socio-demographic factor would explain the utilization of ANC service using odds ratio [13]. The odds ratio is used as a parameter in the logistics regression model to indicate the odds of success (in this case having at least 4 ANC visits) compared to the reference category. The statistical analysis was done using SPSS (version 20) software.

## Results

Among the surveyed 978 women from the three rural zones in Southwest Ethiopia, 430 (44%) of them utilized ANC service for less than four times, and the remaining 548 (56%) women used the ANC service for at least four times during their pregnancy. Percentages of the women who utilized the ANC service during their pregnancy and their socio-demographic category are shown in Tables 1 and 2. According to the percentages in the different categories shown in Table 1, women following Orthodox, Catholic, and Protestant religions used the ANC service at least four times whereas women who follow Muslim and Traditional religions used the ANC service less than four times. When the educational level of the woman and the husband is illiterate, the women made less than four ANC visits; however, they made at least four ANC visits when they completed either elementary or secondary or post-secondary level of education (Table 1). Although women in each of the four occupations made at least four ANC visits, the percentages indicate that at least two-folds of the women in the Trader, Security worker, and Office worker occupation categories made at least four ANC visits (Table 1). The percentages shown in Table 1 indicate that women who had abortion made 1.7 times more visits at least four times than those who made less than four ANC visits. In contrast, those women who did not have abortion made 1.2 times more visits at least four times than those who made less than four ANC visits.

**Table 1:** Percentages of ANC service utilization by women in 8 socio-demographic categories. The numbers shown in each cell are the counts out of n = 978 converted to percentages

Categorical variable	Category	Number of ANC visits (%)	
		<4	≥4
Zone	Kefa	15.3	26.1
	Sheka	2.0	9.4
	Bench Maji	26.6	20.6
Marital status of the woman	Single	1.8	1.9
	Married	36.4	45.7
	Divorced	3.4	6.0
	Windowed	2.4	2.4
Religion	Orthodox	17.2	25.8
	Catholic	1.1	3.1
	Muslim	4.2	4.0
	Protestant	15.6	22.0
	Traditional	5.8	1.2
Educational level of the woman	Illiterate	24.4	21.9
	Elementary	14.0	22.9
	Secondary	5.2	10.1
	Post-secondary	0.3	1.1
Educational level of the husband	Illiterate	19.1	16.3
	Elementary	14.7	23.9
	Secondary	8.8	12.9
	Post-secondary	1.3	3.0
Occupation of the mother	Farmer	39.6	46.4
	Trader	3.4	6.4
	Security worker	0.3	0.5
	Office worker	0.7	2.7
Occupation of the husband	Farmer	38.4	43.0
	Trader	1.7	4.9
	Security worker	3.6	7.7
	Office worker	0.2	0.4
Was there abortion?	Yes	5.6	9.3
	No	38.3	46.7

The percentages shown in Table 2 reveal that more of the women who took iron-folate supplement visited the ANC service at least four times compared to those who did not take iron-folate supplement. Another variable that showed a glaring contrast was source of information where more of the women whose source of information is radio or health professional or relative or partner visited ANC service at least four times compared to those in the traditional midwife and other categories.

**Table 2:** Percentages of ANC service utilization by women in 11 socio-demographic categories. The numbers shown in each cell are the counts out of n = 978 converted to percentages

Categorical variable	Category	Number of ANC visits (%)	
		<4	≥4
Iron-folate supplementation use	Yes	11.6	32.5
	No	32.4	23.5
Decision maker	Mother	4.2	7.1
	Husband	2.1	2.2
	Joint	37.5	46.6
	Other	0.1	0.1
Knowledge of danger signs of pregnancy	Knowledgeable	8.5	27.8
	Not knowledgeable	35.5	28.2
Birth interval (years)	<2	7.7	9.7
	2– 3	26.2	16.7
	3 – 5	7.8	18.5
	≥5	2.4	11.1
Source of information	Radio	1.1	2.9
	Health professional	32.6	41.2
	Traditional midwife	1.7	0.4
	Relative	1.6	3.0
	Partner	0.8	3.7
	Other	6.0	4.9
Volunteering	Yes	4.5	1.5
	No	39.5	54.5
Timely visit	Yes	13.8	49.9
	No	30.2	6.1
Transportation problem	Yes	12.4	15.1
	No	31.6	56.0
Satisfaction of previous visits	Yes	24.7	51.6
	No	19.2	4.4
Distance	< 10.5 km	37.5	43.8
	≥ 10.5 km	6.4	12.3
Age of the woman	15 –19	1.0	1.1
	20 –24	10.6	12.5
	25 –29	13.7	18.6
	30 –34	7.1	10.0
	35 –39	9.0	10.3
	40 –44	2.4	2.5
	45+	0.2	1.0

A substantially higher portion of the women who started the service at the right time had at least four visits, and more of those who did not start their visit at the right time had less than four visits (Table 2). Satisfaction of previous visits was also highly related to the utilization of the ANC service where a vast majority of the women who were satisfied with their previous visits had at least four ANC visits, but a bigger portion of those who were not satisfied had less than four visits (Table 2). According to the percentages shown in Table 2, while slightly more women who live within 10.5 km distance of the service had at least four visits, almost two times more women living more than 10.5 km away from the location of the service had at least four visits.

Table 3 shows the results of the multiple logistic regression model. For the overall population, zone, marital status of the woman, educational level of the husband, occupation of the husband, knowledge of the danger signs of pregnancy, birth interval, source of information, timely visits, and transportation problem were significant factors affecting ANC service utilization. Bench Maji zone has an odds ratio (OR) of 0.374, which implies that compared to Kaffa zone, women have lower chance of making ≥ 4 ANC visits. On the other hand, women in Sheka zone have OR of 1.999, which means they are almost two times likely to make ≥ 4 ANC visits compared to those in Kaffa zone (Table 3).

**Table 3:** Parameter estimates of logistic regression modelon ANC visit utilization (< 4 ANC visits and ≥4 ANC visits) in Kaffa, Sheka, and Bench Majizones

Categorical Variable	Category	Estimate	P-value	Odds ratio	95% CI for Odds ratio
Zone [Ref = Kaffa]	Sheka	0.693	0.124	1.999	(-0.685, 1.955
	Bench Maji	-0.983	<0.001	0.374	(1.422, 6.074)
Marital status of the woman [Ref = Single]	Married	0.913	0.033	2.492	(0.280,3.219)
	Divorced	1.274	0.013	3.576	(0.703, 3.677)
	Widowed	0.882	0.147	2.416	(-1.220, 8.469)
Educational level of the husband [Ref = Illiterate]	Elementary	0.722	0.002	2.058	(1.121, 12.203)
	Secondary	0.670	0.027	1.954	(1.778, 18.048)
	Post-secondary	-0.382	0.577	0.682	(-0.673, 5.669)
Occupation of the husband [Ref = Farmer]	Trader	-0.165	0.566	0.848	(-0.082, 0.548)
	Security worker	2.146	0.048	8.550	(0.087, 0.642)
	Office worker	0.674	0.237	1.962	(-0.987, 64.624)
knowledgeable about the danger signs of pregnancy [Ref = Yes]	No	-1.026	<0.001	0.358	(1.869, 4.083)
Birth interval [Ref <2 years]	2 – 3	-0.051	0.838	0.950	(-0.311, 1.205)
	3 – 5	0.857	0.002	2.355	(0.148, 0.549)
	≥5	0.974	0.019	2.648	(0.375, 1.253)
Source of information [Ref = Radio]	Health professional	-0.846	0.089	0.429	(-0.936, 7.433)
	Traditional midwife	-2.094	0.020	0.123	(0.716, 3.172)
	Relative	0.204	0.751	1.226	(-0.127, 6.167)
	Partner	0.732	0.297	2.080	(-1.872, 14.597)
	Other	-1.819	0.004	0.162	(1.618, 14.076)
Timely visit [Ref = Yes]	No	-2.896	<0.001	0.055	(5.041, 14.493)
Transportation problem [Ref = Yes]	No	-0.648	0.008	1.911	(0.355, 0.916)

The odds ratios of women whose marital status is married, divorced, and widowed are 2.492, 3.576, and 2.416, respectively, which indicate that, compared to single women, married, divorced, and widowed women are 2.494, 3.576, and 2.416 times more likely to make at least four ANC visits. Women whose husband completed elementary **(**OR = 2.058) and secondary (OR = 1.954) school have almost two times chance of making at least four ANC visits, compared to the women whose husband is illiterate. But women whose husband had post-secondary (OR = 0.682) education have less chance of making at least four ANC visits compared to those whose husband is illiterate (Table 3).

Women whose husband’s occupation is security worker have 8.55 times more chance of making at least four ANC visits compared to the women whose husband’s occupation is farmer; and women whose husband’s occupation is office worker are 1.962 times more likely to make at least four ANC visits. On the other hand, women whose husband’s occupation is trader are less likely (OR = 0.848) to make at least four ANC visits than those whose husband’s occupation is farmer (Table 3). Women who do not have knowledge about the danger signs of pregnancy are less likely (OR = 0.358) to make at least four ANC visits compared to those who are knowledgeable. Women whose birth interval is two to three years (OR = 0.95) are almost as likely to make at least four ANC visits as those whose birth interval is less than two years. However, women whose birth interval is three to five years (OR = 2.355) and five or more years (OR = 2.648) are more than twice likely to make at least four ANC visits than those whose birth interval is less than two years (Table 3).

Compared to the women whose source of information is radio, the women whose source of information is a health professional (OR = 0.429), a traditional midwife (OR = 0.123), and other (OR = 0.162) are less likely to make at least four ANC visits; whereas those whose source of information is a relative (OR = 1.226) or a partner (OR = 2.08) are more likely to make at least four ANC visits. These odds ratio values indicate that a partner has the highest positive influence on the success of making at least four ANC visits (Table 3).

Women who did not make a timely visit have a very low chance of making at least four ANC visits (OR = 0.055) compared to those who had. The other significant factor influencing the utilization of ANC service if transportation problem. As shown in Table 3, the women who did not have transportation problem are almost two times (OR = 1.911) more likely to make at least four ANC visits.

## Discussion

Forty-four % of the women who had a five-year or younger child utilized ANC service below WHO’s recommendation, and 56% of the women used the service as recommended by WHO (at least four ANC visits). This implies that lots of women who live in the rural areas of the three zones need to improve their awareness of ANC and commitment to utilize the ANC service to ensure maternal care. This disparity is consistent with what was reported in other parts of Ethiopia [14, 15].

The binary logistic regression analysis revealed that, in the rural area of southwestern Ethiopia, women whose husband completed elementary or secondary education, and women without transportation problem had higher chance of utilizing the ANC service as recommended by WHO than those whose husband is illiterate and those who had transportation problem, which are consistent with what was reported in Menit-Shasha District, Ethiopia [16] and in Addis Ababa, Ethiopia [17]. These findings are also similar to what were reported in other studies including in Nigeria [7], in Kenya [18], in Afghanistan [19], in Arsi zone, Ethiopia [20], in Hadiya zone, Ethiopia [21], in Bahir Dar, Ethiopia [22], in Ethiopia [23], and in Tigray, Ethiopia [24]. This indicates that uneducated husbands make women’s utilization of the ANC service less likely compared to educated husbands in southwestern Ethiopia, which highlights the comparative advantage of husband’s education. Also, in addition to improving socio-demographic attributes and transportation problems, which is a serious hindrance in different parts of Ethiopia such as Afar region [25], husband’s education may give power to women to make informed decisions on things that affect their wellbeing, and this may contribute to increased utilization of healthcare services including the ANC service. Similarly, better-educated husbands’ and availability of transportation facilities would make understanding of the importance of ANC services and accessibility of the services easier.

The contribution of source of information to the odds of ANC utilization was unequally tilted in this study; i.e., the odds ratio of using ANC service by women who got information from a healthcare worker or a traditional midwife was lower than those who got information from radio. These findings are similar to those of a nation-wide data in Ethiopia [23], and from Addis Ababa [17] where they highlighted the important role of media exposure for more ANC service utilization. Although a relative or a partner is more trusted than information obtained via radio, the finding that radio is more effective than healthcare workers and traditional midwives in convincing women to have at least four ANC visits suggest that radio or a similar mass media is a viable platform to disseminate healthcare information. This will also help to get women make a timely first visit, which substantially increases the odds of having at least four ANC visits. The importance of timely first visit was also reported in other studies conducted in Ethiopia [26, 27].

The high odds ratio of having at least four ANC visits by women whose husband’s occupation is security worker, whose birth interval is 3-5 or at least 5 years, and whose marital status is married or divorced or widowed are similar to what were reported in New Zealand [28], Uganda [29], Hadiya Zone of Ethiopia [30], Nepal [31], Menit-Shasha District, Ethiopia [16], Benishangul Gumuz Region of Ethiopia [32], South Africa [33], Botswana [34], and Sidama zone in SNNPR of Ethiopia [35].

## Conclusions

The findings of the study indicated that the source of information, mother’s and husband’s educational level, the zone, previous satisfaction of the service, knowledge of the mother about the danger signs of pregnancy, and birth interval are highly related to the prevalence of ANC service utilization. The multiple logistic regression model analysis revealed that these factors and transportation problems, timely visit of the ANC service, the source of information, and marital status are important factors affecting the likelihood of getting the recommended (at least four) ANC visits during their pregnancy. Therefore, it is recommended to seriously consider these factors when implementing ANC interventions to increase the number of ANC visits during pregnancy to reduce maternal morbidity and mortality of babies.

## Abbreviations

ANC: Antenatal care
CSA: Central Statistical Agency
OR: Odds ratio
SNNPR: Southern Nations, Nationalities, and Peoples' Region
WHO: World Health Organization
